# Contact network analysis of COVID-19 Delta variant outbreak in urban China —based on 2,050 confirmed cases in Xi’an, China

**DOI:** 10.1186/s12889-022-14882-3

**Published:** 2022-12-22

**Authors:** Yang Zhangbo, Chen Zheng, Wang Hui

**Affiliations:** 1 School of Humanities and Social Science, Xi’an Jiaotong University, Xi’an, China; 2 Institute for Empirical Social Science Research, Xi’an Jiaotong University, Xi’an, China; 3 School of Management, Xi’an Jiaotong University, No.28 Xianningxi Road, Xi’an, 710049 China

**Keywords:** COVID-19, Delta variant, Contact network, Interval, Infection route

## Abstract

**Background:**

The purpose of this paper is to study how the Delta variant spread in a China city, and to what extent the non-pharmaceutical prevention measures of local government be effective by reviewing the contact network of COVID-19 cases in Xi’an, China.

**Methods:**

We organize the case reports of the Shaanxi Health Commission into a database by text coding and convert them into a network matrix. Then we construct a dynamic contact network for the corresponding analysis and calculate network indicators. we analyze the cases’ dynamic contact network structure and intervals between diagnosis time and isolation time by using data visualization, network analysis method, and Ordinary Least Square (OLS) regression.

**Results:**

The contact network for this outbreak in Xi’an is very sparse, with a density of less than 0.0001. The contact network is a scale-free network. The average degree centrality is 0.741 and the average PageRank score is 0.0005. The network generated from a single source of infection contains 1371 components. We construct three variables of intervals and analyze the trend of intervals during the outbreak. The mean interval (interval 1) between case diagnosis time and isolation time is − 3.9 days. The mean of the interval (interval 2) between the infector’s diagnosis time and the infectee’s diagnosis time is 4.2 days. The mean of the interval (interval 3) between infector isolation time and infectee isolation time is 2.9 days. Among the three intervals, only interval 1 has a significant positive correlation with degree centrality.

**Conclusions:**

By integrating COVID-19 case reports of a Chinese city, we construct a contact network to analyze the dispersion of the outbreak. The network is a scale-free network with multiple hidden pathways that are not detected. The intervals of patients in this outbreak decreased compared to the beginning of the outbreak in 2020. City lockdown has a significant effect on the intervals that can affect patients’ network centrality. Our study highlights the value of case report text. By linking different reports, we can quickly analyze the spread of the epidemic in an urban area.

## Background

The outbreak of coronavirus disease (COVID-19) pandemic sweeps the world in late 2019 [1–3]. By 2022, different variants of the virus evolved, such as Delta and Omicron, triggering several waves of worldwide infection [4, 5]. Having documented 617 million COVID-19 cases, more than 6.5 million deaths have been reported worldwide by October 2022.

The analysis of existing studies on COVID-19 is mostly based on epidemiology, virology and medicine, involving virus case studies, transmission model construction, gene sequencing, and clinical diagnosis [1, 5–7]. Studies establishing contact networks between confirmed cases based on infection routes are less [8].

Human social network plays a significant role in the spread of viruses. Viruses spread along with human movement and aggregation, thus creating contact networks [9–11]. Several studies have been conducted to analyze the spread of various viruses in populations from a network perspective, such as sexually transmitted diseases [9], AIDS [12], and the Plague [10]. These studies have found that the mode of transmission of different viruses makes the structure of the contact network very different. For example, for HIV through sex, blood and mother-to-child contact, the network structure is simple and sparse [12]. However, for airborne viruses such as novel coronavirus, the network structure is complex [3].

There are some studies based on real-world data describing the contact network at the beginning of the COVID-19 outbreak in different regions, such as China [3], Korea [8], and India [13]. However, these studies only conducted based on data from first wave of the epidemic, when people lack systematic intervention policies, drug treatments and vaccines, and the viruses they analyzed were not emerging delta or omicron variants.

In December 2021, a new wave of an outbreak caused by a Delta variant leaked from a Pakistani flight was reported in Xi’an, China. The outbreak infected more than 2000 people and was the largest COVID-19 outbreak in China since the Wuhan outbreak in 2020. By reviewing this outbreak, we can better know how the Delta variant spread, and to what extent preventive measures be effective.

Based on 2050 confirmed cases reports from December 09, 2021 to January 18, 2022 published by the Shaanxi Provincial Health Commission, we use network analysis techniques to construct and visualize the contact network of cases and calculate the network indicators. The analysis of the contact network is key to understanding the spread of disease [3, 8, 14]. First, based on the network structure, we can clarify the transmission path of the virus and assist in identifying hidden infection paths. Second, based on the diagnosis and isolation dates of pairs of infectors and infectees, we construct three variables of intervals and analyze the trend of intervals during the outbreak, which can help us to evaluate the effectiveness of non-pharmaceutical measures and predict disease trends and health care demands [1, 15]. Third, we also discuss the effect of city lockdown on the intervals and network transmission capacity.

## Methods

### Study population and setting

Xi’an is the largest city in northwestern China and the capital city of Shaanxi Province, with an area of 10,108 km and a resident population of 12,952,900. Xi’an had no large-scale outbreak of COVID-19 before the outbreak in early 2022. The cumulative number of locally confirmed cases before the outbreak was only 263. The cumulative number of infections in this outbreak exceeded 2000 cases.

### Origin of the outbreak in Xi’an

On December 9, 2021, the first case of this outbreak was reported by the Shaanxi Provincial Health Commission. The first infected person was a staff member of the International Passenger Isolation Hotel. Based on full genetic sequencing of the cases, all viruses from patients during this epidemic were Delta variants of SARS-CoV-2, which was highly homologous to the specimen of flight PK854 confirmed passenger entering from Pakistan on December 4. After the outbreak, the local government isolated the confirmed cases and their close contacts, conducted a large-scale universal screening (nucleic acid test), and the city was into lockdown on December 23.

### Data

The database was collected from the daily case reports published by the Shaanxi Provincial Health Commission on its official website, which was collected by a dedicated epidemiological investigation team. The daily reports reported information on cases’ age, sex, residence area,^1^ diagnosis date, isolation date, and their infectors. Data began on December 9, 2021 and ended on January 18, 2022. Since then, there are no new detailed confirmed cases reported. On January 20, 2022, the local government began to reopen the city. The total number of cases is 2050.

### Methods and indicators

We first organized the case reports into a database by text coding. The case reports were in text format, from which we extracted the corresponding variables and assign values. We reconstructed whole contact chains during the disease outbreak by analyzing the infection routes among cases. The raw data of transmission routes between confirmed cases were converted into a symmetrical square matrix (2050 × 2050). For example, if there is a text report that case A is an infector of case B, the value of row A and column B in the matrix is 1, otherwise 0. We visualized the matrix as a network where nodes were confirmed cases, ties were the infection routes between them.

The dynamics of the network have an important impact on the risk of infection, and the structure of dynamic networks is very different from that of static networks [16, 17]. Therefore, we construct a dynamic contact network for the corresponding analysis. Then, we visualize the dynamic network and calculate network indicators as follows.

#### Degree centrality

It indicates the number of direct contacts of a case. This indicator has a high correlation with the virus’s basic generation number and can be used to measure the rate of transmission [18]. The formula is as follows, where *i* is the case, *j* is his contact and *n* is the network size. The *a* is the matrix where *i* and *j* are the elements.

$${C}_D(i)=\sum_{j=1}^n{a}_{ij}$$

#### PageRank score

PageRank algorithm considers both the number of direct contacts and indirect contacts of the diagnosed case through an iterative settlement [19]. Mathematically it is expressed as:

$${\textrm{PR}}_i=\sum_{j\in {B}_i}\frac{{\textrm{PR}}_j}{n_j}$$

where PR_*j*_ is the PageRank value of the case *j*’s direct tied with the case *i*, *n*_*j*_ indicates the number of cases directly connected to case *j*, *B*_*i*_ is the set of all nodes linked to the focal node j. The initial PageRank value is distributed equally to each actor based on the size of the network. The PageRank value on the right side of the equation is the PageRank value of the previous iteration. Since the PageRank score takes into account not only direct ties but also indirect ties, the value is more reflective of the patient’s position in the whole network [20]. The higher the PageRank score of a patient takes, the higher its risk of spreading the virus in the overall transmission network [3].

#### Component number

Community detection is a key problem in graph mining. Its main purpose is to partition the network into different subgraphs [21]. We use components to divide the contact network into clusters or groups that have a high degree of internal cohesion and a low degree of external cohesion between different clusters. A component is a set of nodes where any two nodes have a connected path, while there is no path between any two components [22].

#### Triadic census

**Fig. 1:**
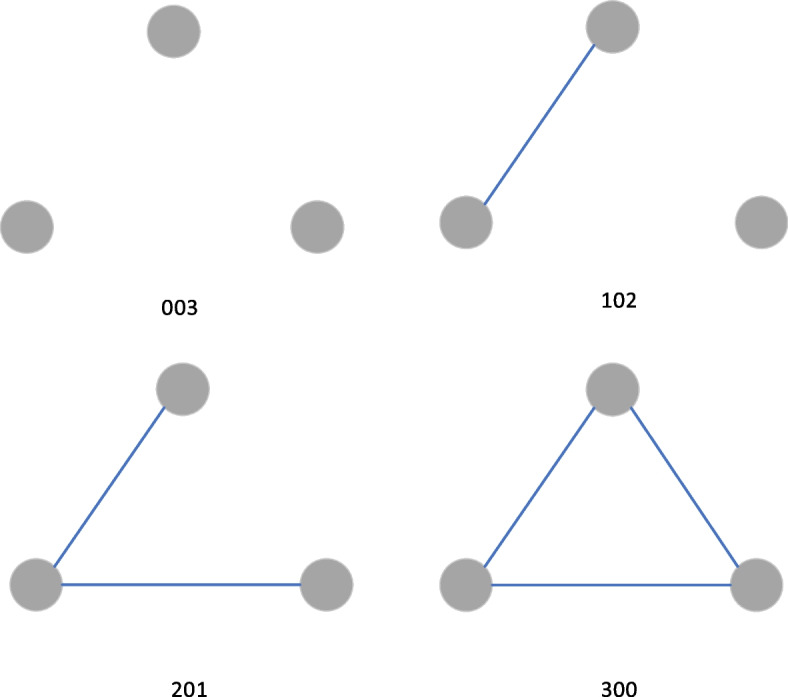
Examples of the triadic census in a network

We also count the number of different triadic structures in the network. The motifs of three nodes in a network are the basic units of the network [23]. The triadic census is a useful method to explore the micro-level structure of a network [24]. Analysis of subgraphs of three nodes (triads) is just well suited for examining social interactions in epidemiology since triads can reflect the micro-level spread pattern of an epidemic. For example, in a “linear” transmission pattern, all relations are one-directional, which is like case A transmitted to case B, B transmitted to case C, and A did not have direct contact with C [25]. Since contact networks are undirected networks, there are four basic triadic structures, 003 (no contact relations between the three actors), 102 (only one relationship between three actors), 201 (two contact relationships among three actors), 300 (full connected triadic) as shown in Fig. 1.

#### Intervals

Existing studies often use serial intervals to analyze the risk of disease transmission. The COVID-19 serial interval is the time interval between the onset of symptoms in a primary (infector) case and that of a secondary (infected) case [1, 15].

However, because many cases were isolated before symptoms appeared, case reports do not show the time of symptoms’ onset. Based on the infection routes, we construct three intervals and calculate their distributions. Interval 1 is the time duration between the time of diagnosis and the time of isolation of a case. Interval 2 is the time duration between an infector diagnosis time and infectee diagnosis time. Interval 3 is the time duration between an infector isolation time and an infectee isolation time which is the date that a confirmed case was isolated for being a contact.

We use Ordinary Least Square (OLS) regression models to test how intervals affect the transmission level of an infectee. The regression formula is expressed as:

$$\textrm{Y}=\upbeta 0+\upbeta 1\textrm{Interval}+\textrm{Control}+\upvarepsilon$$

where Y is the dependent variable (degree centrality and PageRank score), Interval is the independent variable, Control variables include gender, age, severity of symptom, and resident places^1^ that extracted from case reports. In total, 2050 samples entered into the regression analysis. One of the cases did not report age, which we coded as missing value. Intervals also had some missing values because some cases did not report the date of diagnosis and isolation.

### Ethics

Ethical approval for this study was received from the Ethics Committee of Xi’an Jiaotong University Health Science Center (No. 2020–1217), and all methods were performed in accordance with the relevant guidelines and regulations. We anonymized all data.

## Results

The mean age of cases is 35.90 years with a median of 34 years. The mean degree centrality is 0.74 and the maximum number of contacts is 43. The mean value of the degree less than 1 represents the end of the outbreak. It means that one patient in the network of this outbreak is in contact with an average of 0.74 confirmed cases. The maximum value of degree centrality indicates that this outbreak had one case linked with 43 other confirmed cases. The mean PageRank score is 0.0005. At the beginning of the outbreak in 2020, the average degree centrality of eight regions in China was 1.136 and the average PageRank score was 0.0093 [3]. There is slightly more male with a percentage of 54.54%. Most cases (94.19%) have a mild symptom. Eight cases are asymptomatic infections. The results of descriptive statistics are presented in Table 1.

**Table 1:** Descriptive statistics of Xi’an COVID-19 cases

Variables	N	Mean	Median	S.D.	Min	Max
Age	2049	35.90	34	17.70	4 days	94
Degree centrality	2050	0.741	1	1.379	0	43
PageRank score	2050	0.0005	0.0004	0.0006	0.0001	0.0165
Interval 1	1483	−3.912	−3	3.427	−20	1
Interval 2	730	4.199	3	3.129	0	21
Interval 3	339	2.976	2	3.131	−8	17
**Gender**		**Male**	**Female**			
	2050	1118 (54.54%)	932 (45.46%)			
**Disease severity**		**Asymptomatic infections**	**Moderate**	**Mild**		
	2048	8 (0.39%)	111 (5.42%)	1929 (94.19%)		

Interval 1: the time between diagnosis time and isolation time. Interval 2: the time between the infector’s diagnosis time and the infectee’s diagnosis time. Interval 3: the time between infector isolation time and infectee isolation time

### Network visualization

**Fig. 2:**
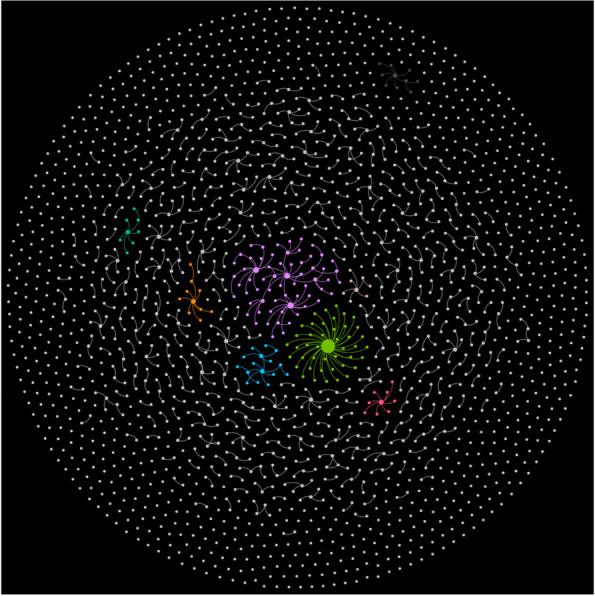
Xi’an COVID-19 contact network (December 9, 2021 - January 18, 2021)

Figure 2 shows the contact network of COVID-19 in Xi’an from December 9, 2021 to January 18, 2022.^2^ The nodes in the figure represent confirmed cases and the edges represent the contact relationships between them. A larger size indicates more cases in contact with the focal case. There are 2050 cases in the network and only 759 edges, making the network very sparse, with a density close to 0. More than 900 components have only one node. The epidemiological investigation by the local government did not find some transmission route between confirmed cases that resulted in the isolated nodes pattern in Fig. 2. After deleting the isolated nodes, we recalculated the network metrics. The average degree centrality is 1.399 and the average PageRank score is 0.0009.

**Fig. 3:**
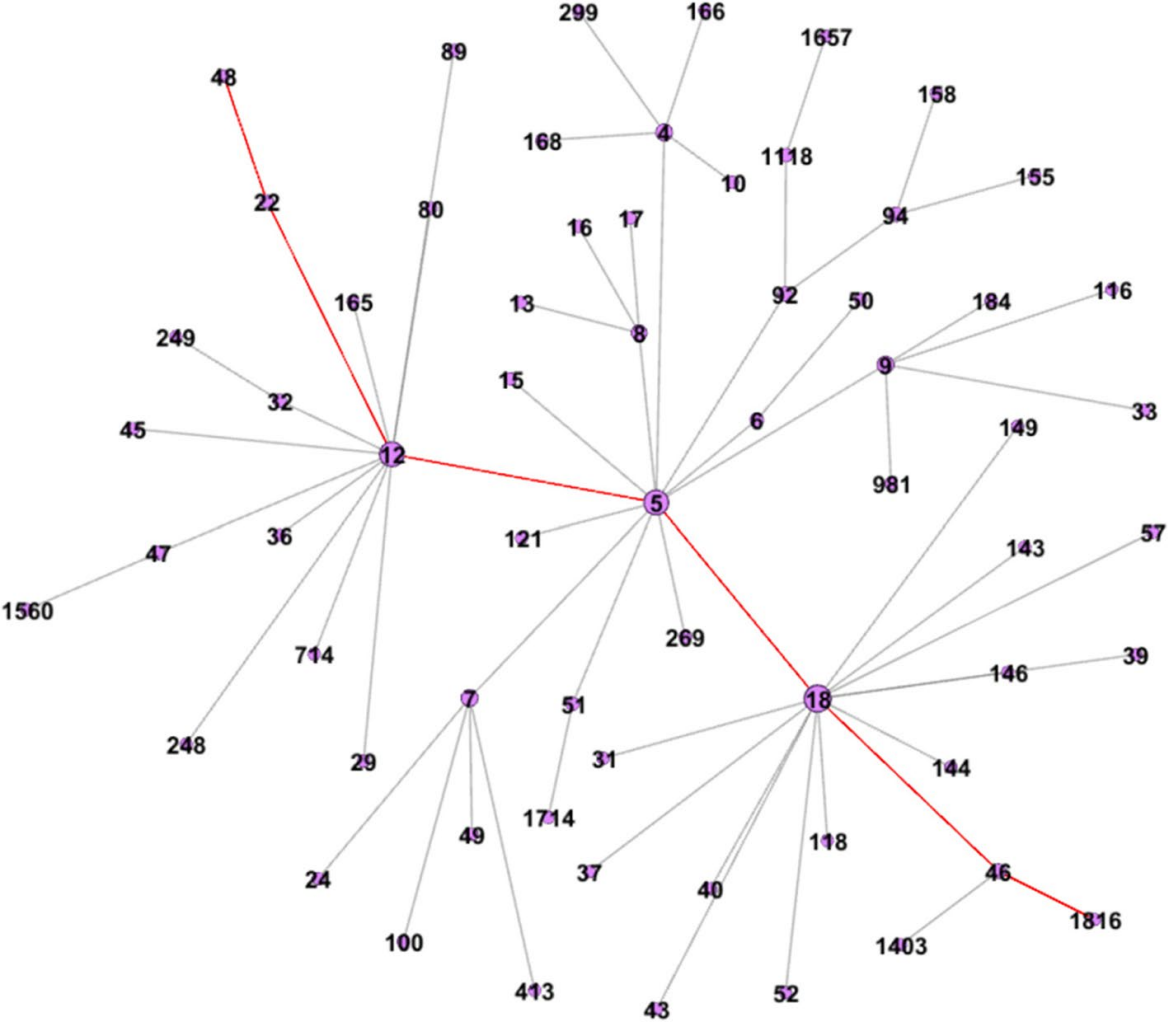
The largest component of the contact network of the COVID-19 outbreak in Xi’an (December 18, 2021 - January 18, 2021)

Figure 3 shows the largest component extracted from the overall network, which is the largest transmission chain during the epidemic. The number of each node is the numeral order, a smaller number means the case was detected earlier. This component contains 64 confirmed cases (3.12% of all cases) and 63 contact relationships (8.3% of all contact relationships). The longest chain of infection in the network has 6 steps, we marked one of them in red color. Based on the dates of the case reports, we speculate that case 5 in this chain first infected case 12 and case 18, followed by case 12 and case 18 then infected other cases, respectively. The node with the highest degree centrality is a staff member of a local university, diagnosed on December 18, 2021. This case went to several shopping malls in Xi’an, thus triggering a mass transmission.

### Network indicators

**Fig. 4:**
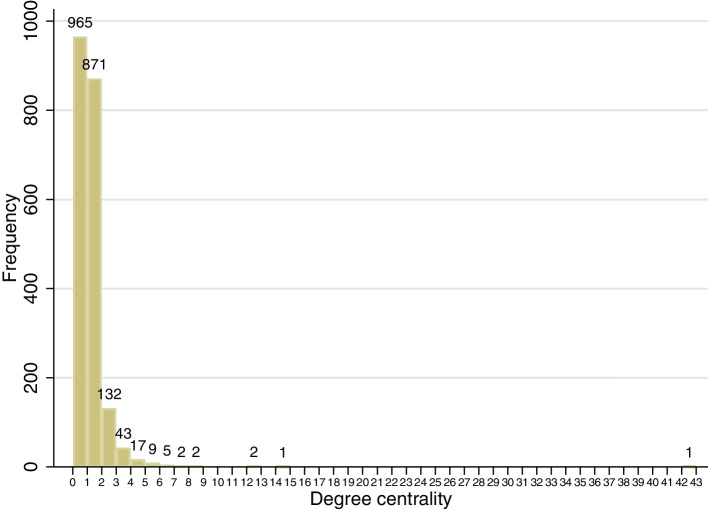
Distribution of degree centrality of Xi’an contact network (*n* = 2050)

Figure 4 demonstrates the distribution of degree centrality for all cases in Xi’an, showing a right-skewed distribution. This means most cases have very few contacts. There are only three super-spreaders (number of transmissions > 10). The degree in this figure indicates the quantity of other confirmed patients that each patient has contact with.

**Fig. 5:**
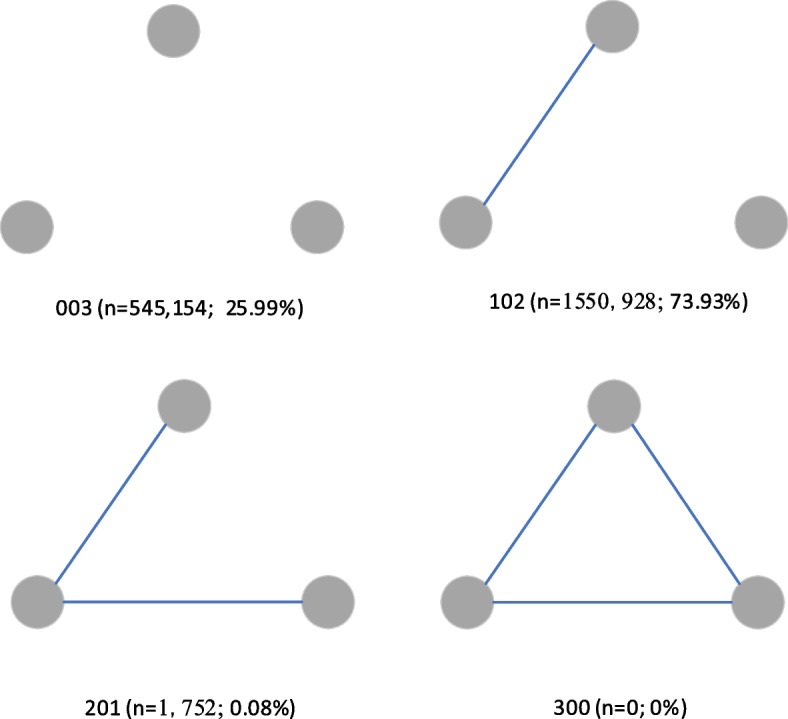
Triad census of Xi’an contact network

The triad census is shown in Fig. 5. The percentage of no contact relationship between the three actors is 25.99% (*n* = 545,154). The percentage of only one relationship between the three actors is 73.93% (*n* = 1,550,928). The percentage of two contact relationships among three actors is 0.08% (*n* = 1752), and the percentage of full connected triadic is 0% (*n* = 0). The triad census implies that the network is sparse, with the vast majority of the motifs containing only one or even no contact relations. Combined with the large number of isolated nodes, we speculate that there are many hidden contagion paths that have not been detected by epidemiological investigation.

### Intervals analysis

**Fig. 6:**
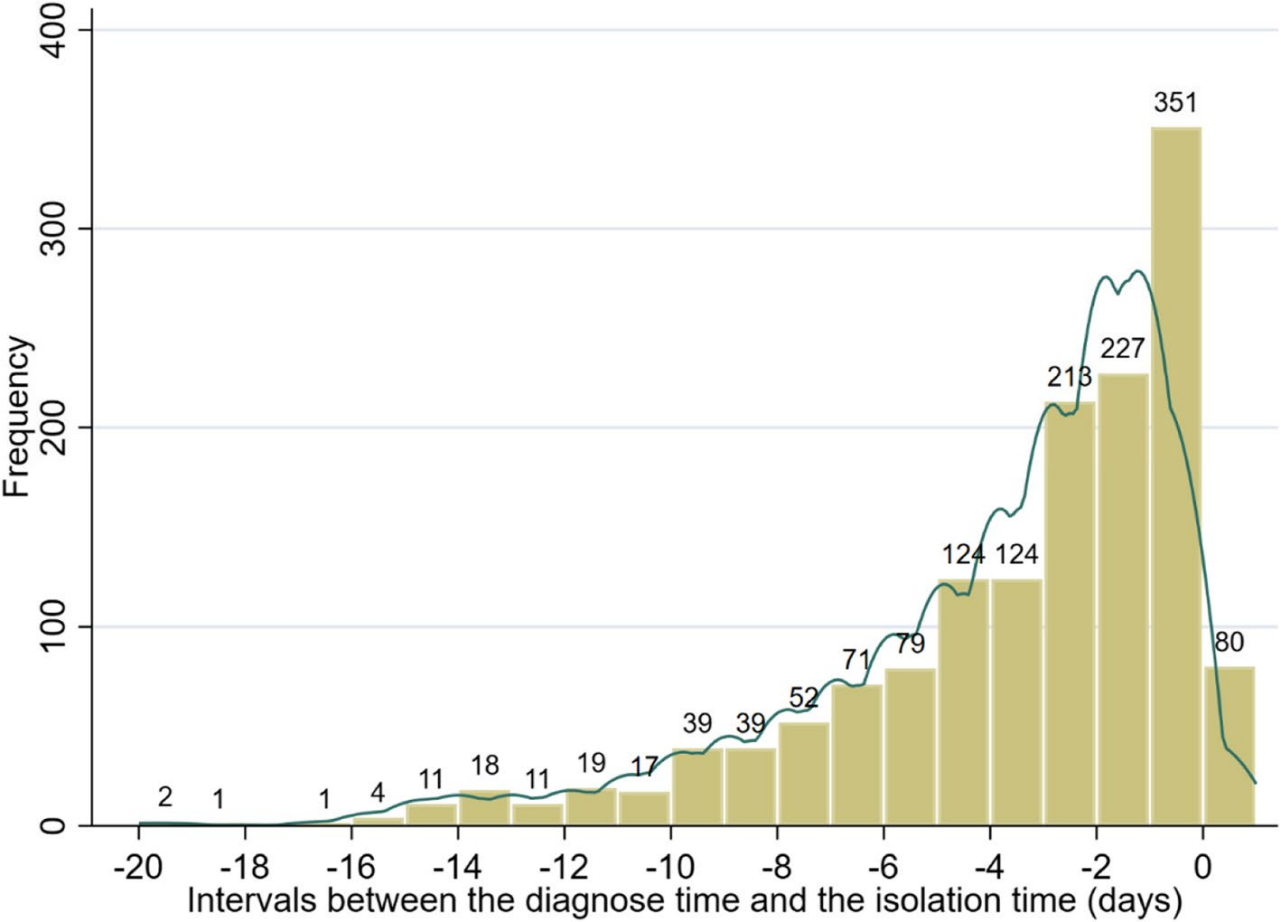
Distribution of intervals between the diagnosis time and the isolation time of confirmed cases (Interval 1)

Figure 6 shows the distribution of intervals between case diagnosis time and isolation time. The mean value is − 3.91 days, with a minimum value of − 20 days and a maximum value of 1 day.

**Fig. 7:**
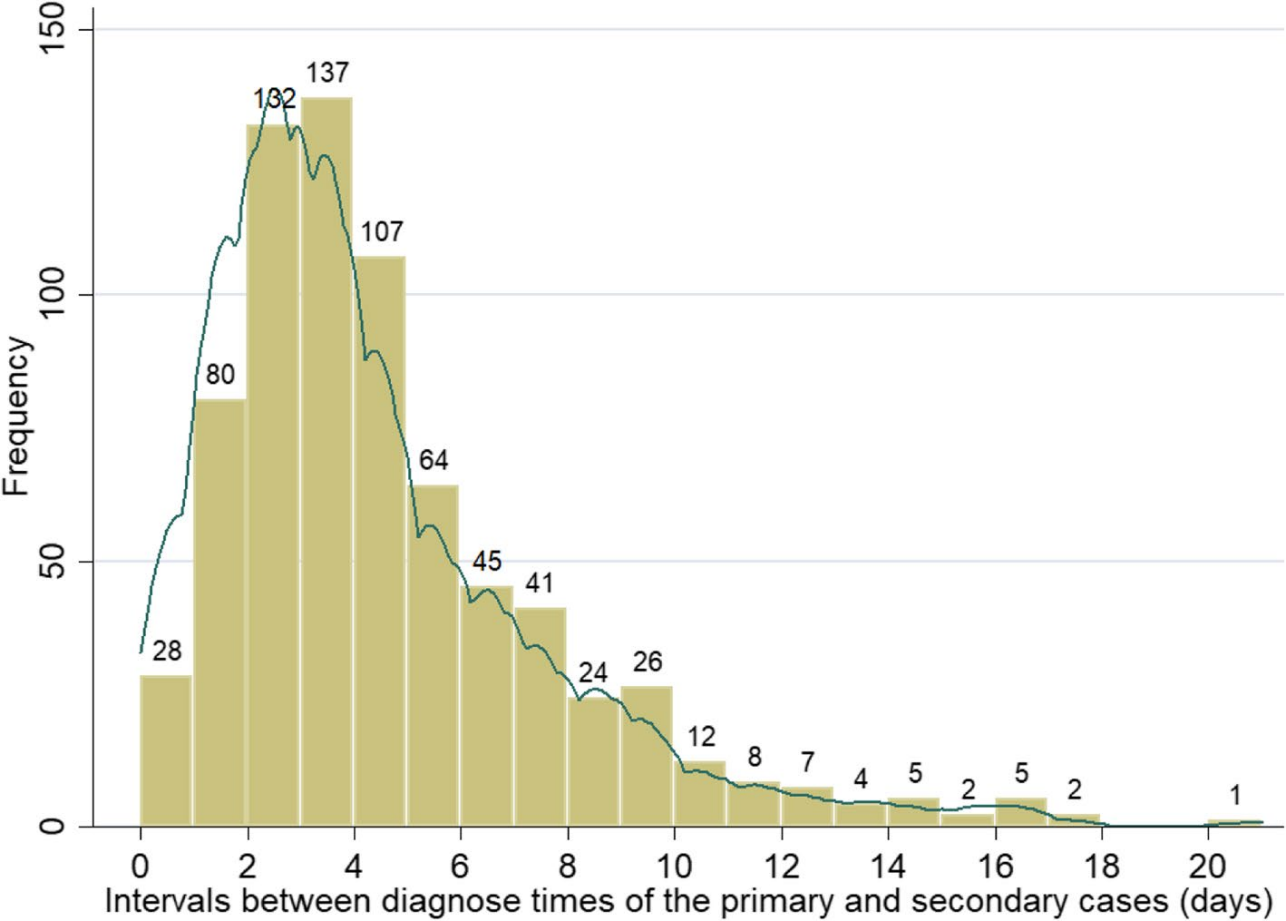
Distribution of intervals between the diagnosis time of the primary case and the diagnosis time of the second case (Interval 2)

Figure 7 shows the distribution of intervals between the diagnosis time of the primary case and the diagnosis time of the secondary case. In general, the main distribution ranges between 0 and 21 days. The mean value is 4.20 days, meaning the infectee is diagnosed 4 days on average after the previous case was diagnosed.

**Fig. 8:**
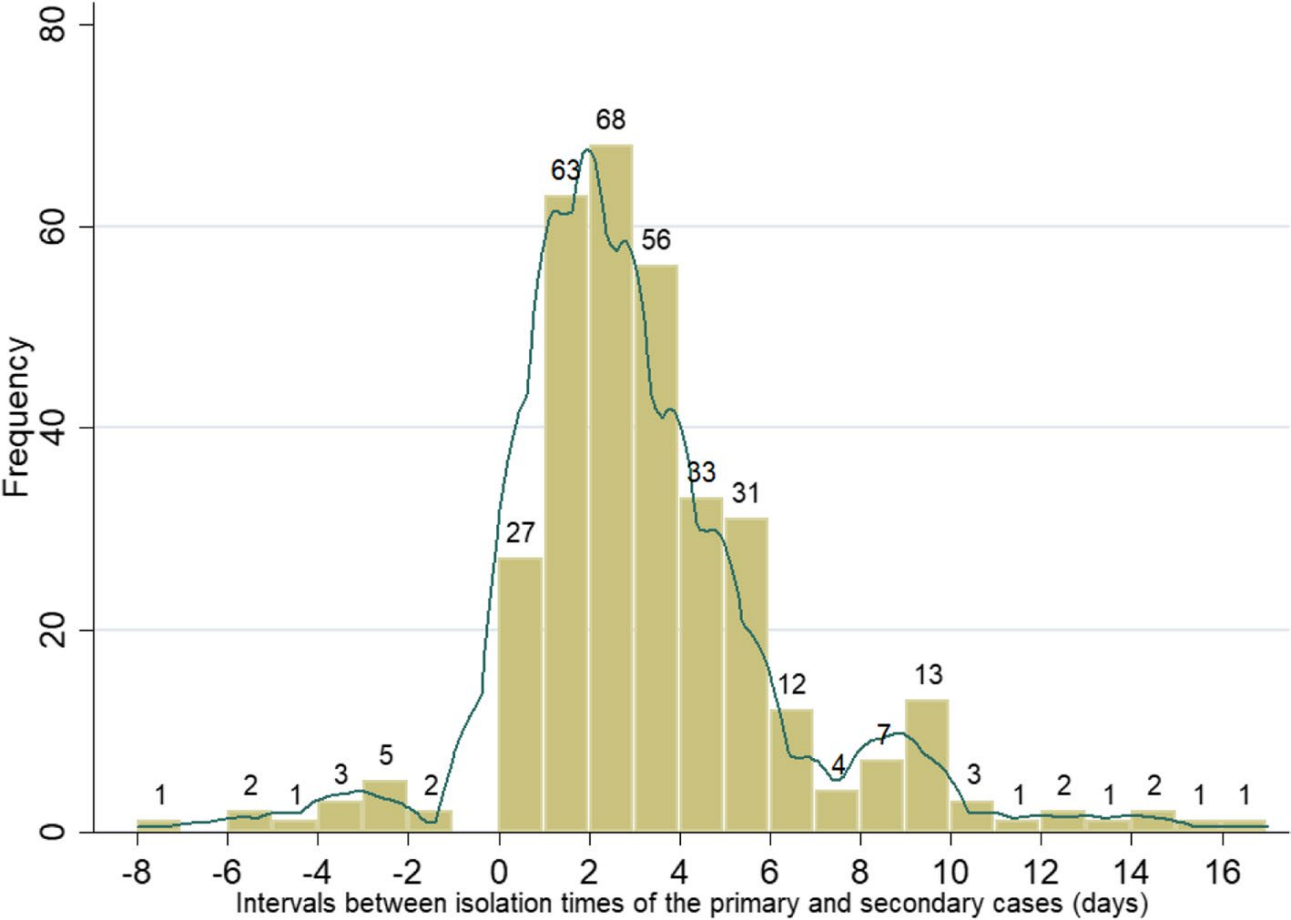
Distribution of intervals between the primary case isolation time and the secondary case isolation time (Interval 3)

Figure 8 illustrates the distribution of intervals between the primary case isolation time and the secondary case isolation time. The overall distribution ranged from − 8 days to 17 days, with a mean of 2.98 days, indicating that a case is isolated for an average of about 3 days after his or her infectors were isolated.

**Fig. 9:**
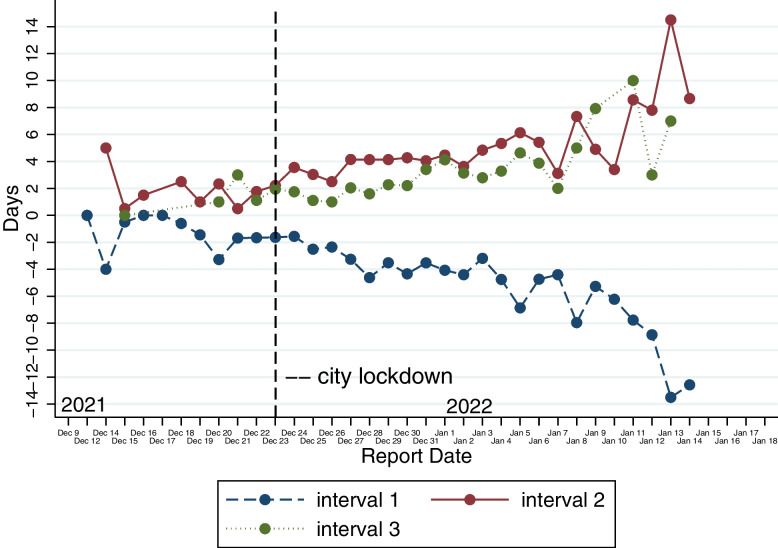
The trend of daily average intervals during the epidemic. Interval 1: the time between diagnosis time and isolation time. Interval 2: the time between the infector’s diagnosis time and the infectee’s diagnosis time. Interval 3: the time between the infector isolation time and the infectee’s isolation time

The trend of the average daily intervals of confirmed cases is shown in Fig. 9. We calculate the three intervals for each case per day based on the daily case reports and then average each of the three intervals for each day to obtain each node in the figure below. There is a downward trend in the negative differential between diagnosis dates and isolation dates (Interval 1). Interval 2 has an upward trend, indicating a gradual increase in the intervals between the infectors’ diagnosis time and the infectees’ diagnosis time. Interval 3 has the same trend as interval 2.

We further compare whether there is a change in interval 1, interval 2 and interval 3 before and after the city lockdown by t-test. Results show that interval 1 declines significantly after the lockdown (− 1.83 days vs. -4.06 days, *p* < 0.001). Interval 2 increases significantly after the lockdown (1.77 days vs. 4.34 days, *p* < 0.001), as well as interval 3 increases significantly after the lockdown (1.58 days vs. 3.04 days, *p* < 0.05). These results still held after we excluded cases on the day of the city lockdown, 1 day after the city lockdown and 2 days after the city lockdown. This suggests that these statistical findings are robust.

Table 2 shows the results of the OLS regressions, in which the number of contacts (degree centrality) is the dependent variable. Model 1 is the baseline model with only control variables, while models 2, 3, and 4 include interval 1, interval 2, and interval 3, respectively. Among the three intervals, only interval 1 has a significant positive effect on degree centrality, while the coefficients of intervals 2 and 3 are insignificant. Because the value of interval 1 was mostly negative, the coefficient indicates that the longer the case was isolated before diagnosis, the fewer people he/she infected. Compared with model 1, the R-square of models 2, 3, and 4 are all improved to some extent, indicating that models with added independent variables have better explanatory power.

**Table 2:** OLS regression results, DV = degree centrality

Variables	Model 1	Model 2	Model 3	Model 4
Age	0.000146	−0.00000354	0.0000555	0.00119
	(0.00176)	(0.00131)	(0.00146)	(0.00110)
Gender (male = 0)	0106	0.0485	0.0591	0.00657
	(0.0621)	(0.0461)	(0.0556)	(0.0409)
Moderate disease (0 = Asymptomatic)	0.815^*^	0.615	1.263	0
	(0.363)	(0.441)	(0.817)	(.)
Mild disease (0 = Asymptomatic)	−0.114	0.143	0.297	− 0.00711
	(0.351)	(0.431)	(0.809)	(0.117)
Interval 1		0.0446^***^		
		(0.00812)		
Interval 2			−0.0233	
			(0.0129)	
Interval 3				0.0120
				(0.00800)
Residential areas	controlled	controlled	controlled	controlled
Constant	0.107	0.465	−0.279	0.941^*^
	(1.051)	(0.964)	(1.142)	(0.374)
*N*	2036	1480	727	337
*R*^2^	0.029	0.062	0.085	0.073

Standard errors in brackets, * *p* < 0.05, ** *p* < 0.01, *** *p* < 0.001. Interval 1: the time between diagnosis time and isolation time. Interval 2: the time between the infector’s diagnosis time and the infectee’s diagnosis time. Interval 3: the time between the infector isolation time and the infectee’s isolation time

Table 3 presents the results of the OLS regression, in which the PageRank score is the dependent variable. Model 1 is the baseline model with only control variables, and interval 1, interval 2 and interval 3 are added to models 2, 3 and 4, respectively. The effect of interval 1 on the PageRank score is significantly positive, indicating that the longer a case is isolated before diagnosis, the fewer direct and indirect contacts he or she has. The effect of interval 2 on the PageRank score is significantly negative, indicating that the longer the interval between diagnosis times of the case and his/her infectee, the lower the PageRank score of the case. The regression coefficient of interval 3 is positive but insignificant. Relative to the baseline model, the R square of the latter three regression models all improved, indicating an increase in the explanatory power of these models.

**Table 3:** OLS regression results, DV = PageRank score

Variables	Model 1	Model 2	Model 3	Model 4
Age	0.000000474	−0.000000252	0.000000316	0.000000313
	(0.000000738)	(0.000000598)	(0.000000574)	(0.000000560)
Gender (male = 0)	0.0000436	0.0000218	0.0000231	0.0000185
	(0.0000260)	(0.0000211)	(0.0000219)	(0.0000209)
Moderate disease (0 = Asymptomatic)	0.000398*	0.000320	0.000621	0
	(0.000194)	(0.000240)	(0.000373)	(.)
Mild disease (0 = Asymptomatic)	−0.0000190	0.000105	0.000340	0.0000545
	(0.000188)	(0.000235)	(0.000369)	(0.0000695)
Interval 1		0.0000208^***^		
		(0.00000308)		
Interval 2			−0.0000110^**^	
			(0.00000356)	
Interval 3				0.00000225
				(0.00000335)
Residential area	controlled	controlled	controlled	controlled
Cons	0.000440	0.000587	0.000283	0.000785^***^
	(0.000613)	(0.000446)	(0.000408)	(0.000187)
*N*	2036	1480	727	337
*R*^2^	0.027	0.064	0.081	0.106

Standard errors in brackets, * *p* < 0.05, ** *p* < 0.01, *** *p* < 0.001. Interval 1: the time between diagnosis time and isolation time. Interval 2: the time between the infector’s diagnosis time and the infectee’s diagnosis time. Interval 3: the time between the infector isolation time and the infectee’s isolation time

## Discussion

The mean value of the degree centrality of the contact network is less than 1, indicating that the outbreak was effectively controlled through isolation and mass screening [11]. Because a large number of infection chains are not fully clarified, the contact network for this outbreak in Xi’an is very sparse, with a density of less than 0.0001, compared to 0.004 for the same area in early 2020 [11].

Based on a report from Shaanxi Health Commission,^3^ this outbreak came from a single source, so its component number should be one. But the components we find are much more than one. There are two possible reasons: the first is that there is a large hidden chain of transmission that was not detected; the second possibility is that the source of this outbreak is not just offshore, but that multiple sources exist, which is an indication that the outbreak existed long before the first case was reported on December 9, 2020. Since the data limitation, it needs further genetic sequencing to clarify this question.

Because of the very large difference in network size between the initial 2020 outbreak and the current outbreak (234 confirmed cases vs. 2050 confirmed cases), the number of components cannot be directly compared. To compare the level of modules, we calculated the modularity scores of two contact networks by using the Louvain method [26, 27]. The modularity value of the 2020 contact network (0.95) and the modularity value of the current epidemic (0.99) are similar, indicating a more consistent level of differentiation of the networks in the two different periods.

**Fig. 10:**
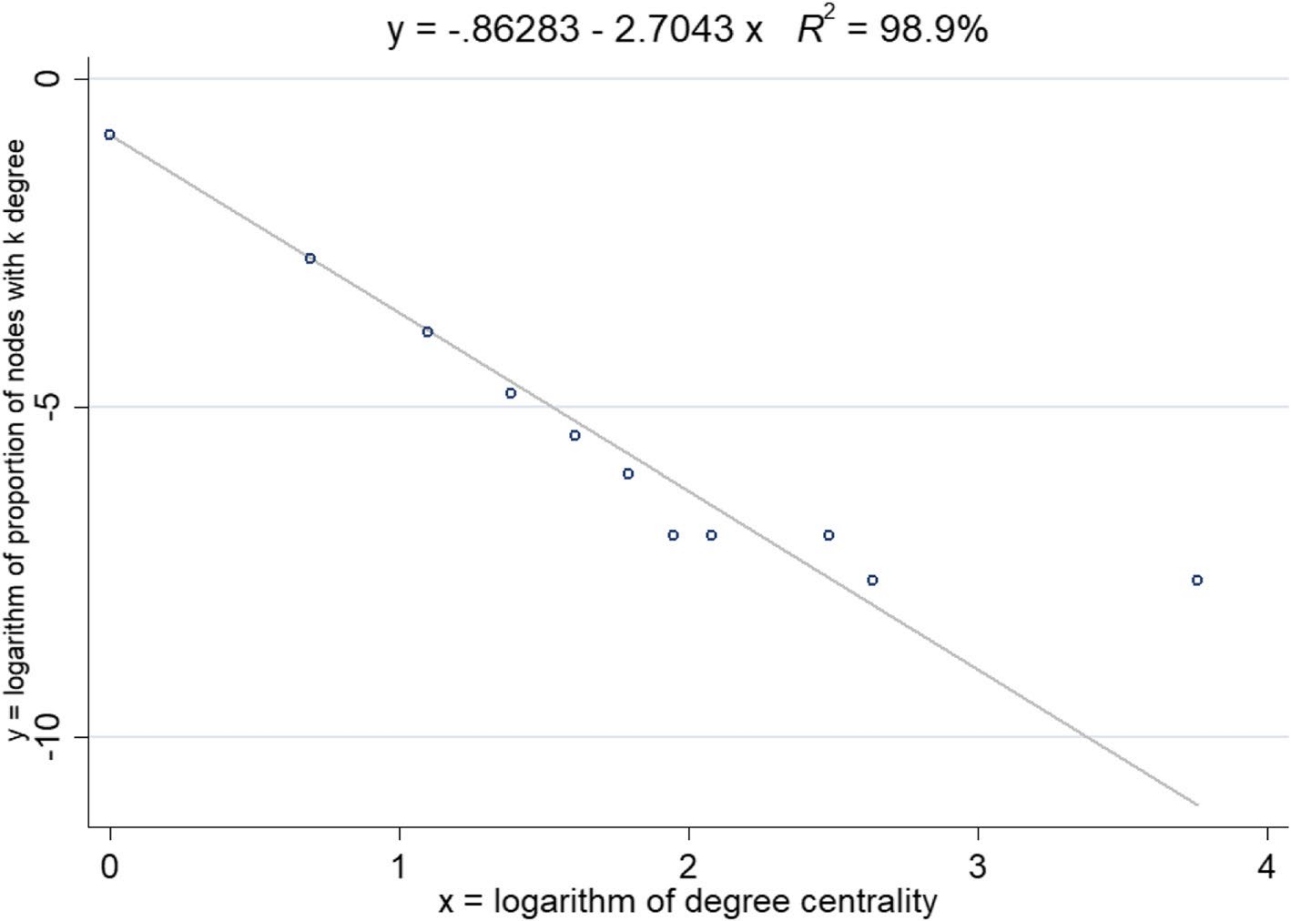
Distribution of degree centrality in logarithmic coordinates

A power-law distribution would form a scale-free network [28]. We fit the distribution of degree centrality to determine whether the distribution of this outbreak is a power-law distribution (*aaplot* program in Stata). The results of the fit are shown in Fig. 10. The fitted line is a straight line after taking the logarithm form, with an intercept term of − 0.86, a slope of − 2.70, and an R square of 98.9%, indicating that the distribution of degree centrality shows a power-law distribution. This indicates that the contact network is a scale-free network, which is consistent with the previous analysis of the contact network in Seoul, Korea [8].

Scale-free networks imply that a few cases have more contacts and most cases have fewer contacts. These super-disseminators in this kind of network accelerate the spread of the virus [29]. For example, the key nodes for the spread of Ebola are these super-dispersers [29]. In such a network, the virus can quickly spread to the entire connected fraction and cause an outbreak even if each node is exposed to a limited number of other nodes [9]. On the other hand, such a network is structurally vulnerable, i.e., by removing a few nodes with a high degree of centrality from the network, the connectivity of the network is drastically reduced. By isolating hubs of contact networks, a network structural break can be caused, and virus transmission can be effectively controlled [9].

By analyzing the diagnosis and isolation time of the network infection chains, we find that the mean value of the intervals (interval 1) between the diagnosis date and isolation date of patients is − 3.9 days. The longest interval is only 1 day. This distribution indicates that the vast majority of cases were isolated before diagnosis based on their contact tracing information. A simulation study using data analysis and epidemiological interventions in four Nordic countries found that if the mean time interval between symptom onset and isolation was reduced from 12 to 4 days, it resulted in an 85.2% reduction in infections and 88.8% reduction in deaths [30]. This suggests that the intervention strategy of this outbreak in terms of reducing interval was effective. In our observation sample, most cases tested positive for nucleic acid even during the isolation period. Figure 9 and the t-test also show that interval 1 showed a decreasing trend after the city lockdown, which indicates that as various measures such as lockdown and universal nucleic acid test progressed, more and more cases were diagnosed in isolation.

The mean value of the time intervals (interval 2) between the diagnosis time of the infector and the time of the infectee is 4.2 days. For comparison, analysis of data from early 2020 of the COVID-19 outbreak in China showed a serial interval of 7.8 days in the early stage and 2.6 days in the later stage [1]. However, vaccines and well-established contact tracing techniques were not available at that time. The trend in Fig. 9 and the t-test we performed suggest that the city lockdown did not have an impact on reducing interval 2. For reference, Hu et al. (2021) found that reducing the delay in implementing an effective detection and follow-up program from 50 days to 10 days reduced infections and deaths by 35.2 and 44.6% respectively [30]. This implies that the local health department should adopt more effective interventions during the outbreak, especially contact tracing, to reduce the interval between the infector’s diagnosis time and the infectee’s diagnosis time.

The mean value of intervals (interval 3) between the isolation time of the infector and infectee is 2.9 days. The smaller the number, the more efficient the public health department is in identifying and isolating the close contacts of confirmed cases and cutting off the source of infection. A negative value indicates that the infectee had been isolated before the infector was isolated. Figure 9 and the t-test we performed illustrate that the city lockdown did not reduce the isolation intervals. Possibly, it is because most later cases are those people with long incubation periods. In summary, our results demonstrate that city lockdowns mostly stop the further spread of the virus but hardly enhance the efficiency of confirming and tracking those already infected people.

The regression results indicate that the shorter interval of the time to diagnosis and isolation of the case (interval 1), the weaker the transmission capacity of the case. Therefore, effective and timely contact tracing and isolation measures are efficient non-pharmacological interventions. The shorter the interval between the diagnosis time of the infector and infectee (interval 2), the higher the whole network centrality (PageRank score) of the infectee. We speculate that this may be due to sample selection bias. The infectee that was quickly tracked and diagnosed was most likely already in a relatively large chain of infection and therefore had more direct and indirect contacts.

## Conclusion

We constructed a dynamic contact network between infector and infectee based on 2050 confirmed cases of COVID-19 (Delta variation) in Xi’an by end of 2021. The network is a scale-free network with an average degree centrality of 0.741. The network contains 1291 components, and the largest component contains 64 cases and 63 infection routes. There are multiple infection routes that were not detected in this outbreak. The mean interval (interval 1) between case diagnosis time and isolation time is − 3.9 days. The mean of the interval (interval 2) between the infector’s diagnosis time and the infectee’s diagnosis time is 4.2 days. The mean of the interval (interval 3) between infector isolation time and infectee isolation time is 2.9 days. After the city lockdown, interval 1 decreases, and interval 2 and interval 3 increase.

There are some limitations to our work. First, it lies in the fact that the epidemiological survey did not fully reveal all infection routes, so the contact network of our study is only a partial reflection of the real transmission network. Second, this contact network is only based on data from one area in China. Data from more areas can be integrated in the future for multi-regional comparison.

## Data Availability

All data and code are on an OSF data repository, see https://osf.io/e4arq/. All raw data (in Chinese) is available on the official website of the Health Committee of Shaanxi Province of China, see http://sxwjw.shaanxi.gov.cn/sy/wjyw/. All the data are anonymous.

## Abbreviations

COVID-19: Corona Virus Disease 2019
OLS: Ordinary Least Squares
STATA: Software for Statistics and Data Science
Aaplot: Aaplot is a Stata module for scatter plot for yvar versus xvar with linear and/or quadratic fit superimposed.

## Footnotes

1 The residential area is based on the residential building of 18 districts of Xi’an, accurate to the building where the individual lives, but not to the floor or room.

2 We also make an animation of the dynamics of the contact network based on case-reported dates, see https://youtu.be/tuXljalE0_k

3
http://sxwjw.shaanxi.gov.cn/sy/wjyw/202112/t20211209_2203443.html

## References

[1] Ali ST, Wang L, Lau EH, Xu X, Du Z, Wu Y (2020). Serial interval of SARS-CoV-2 was shortened over time by nonpharmaceutical interventions. Science.

[2] McCloskey B, Zumla A, Ippolito G, Blumberg L, Arbon P, Cicero A (2020). Mass gathering events and reducing further global spread of COVID-19: a political and public health dilemma. Lancet.

[3] Yang Z, Zhang J, Gao S, Wang H (2022). Complex contact network of patients at the beginning of an epidemic outbreak: an analysis based on 1218 COVID-19 cases in China. Int J Environ Res Public Health.

[4] Burki TK (2021). Lifting of COVID-19 restrictions in the UK and the Delta variant. Lancet Respir Med.

[5] Collie S, Champion J, Moultrie H, Bekker L, Gray G. Effectiveness of BNT162b2 vaccine against omicron variant in South Africa. N Engl J Med. 2021:1–3 https://www.nejm.org/doi/10.1056/NEJMc2119270. 10.1056/NEJMc2119270 PMC8757569 34965358

[6] Chan JF, Yuan S, Kok K, Chu H, Yang J, To KK (2020). A familial cluster of pneumonia associated with the 2019 novel coronavirus indicating person-to-person transmission: a study of a family cluster. Lancet.

[7] Phan LT, Nguyen TV, Luong QC, Nguyen TV, Nguyen HT, Le HQ (2020). Importation and human-to-human transmission of a novel coronavirus in Vietnam. N Engl J Med.

[8] Jo W, Chang D, You M, Ghim G (2021). A social network analysis of the spread of COVID-19 in South Korea and policy implications. Sci Rep-UK.

[9] Bearman PS, Moody J, Stovel K (2004). Chains of affection: The structure of adolescent romantic and sexual networks. Am J Sociol.

[10] Marvel SA, Martin T, Doering CR, Lusseau D, Newman ME. The small-world effect is a modern phenomenon. preprint arXiv. 2013:1310.2636. 10.1002/scin.2008.5591731914.

[11] Yang Z (2021). Analysis of dynamic contact network of patients with COVID-19 in Shaanxi Province of China. Sci Rep-UK..

[12] Jaffe HW (2008). The early days of the HIV-AIDS epidemic in the USA. Nat Immunol.

[13] Azad S, Devi S (2020). Tracking the spread of COVID-19 in India via social networks in the early phase of the pandemic. J Travel Med.

[14] Eubank S, Guclu H, Kumar VA, Marathe MV, Srinivasan A, Toroczkai Z (2004). Modelling disease outbreaks in realistic urban social networks. Nature.

[15] Du Z, Xu X, Wu Y, Wang L, Cowling BJ, Meyers LA (2020). Serial interval of COVID-19 among publicly reported confirmed cases. Emerg Infect Dis.

[16] Armbruster B, Wang L, Morris M (2017). Forward reachable sets: Analytically derived properties of connected components for dynamic networks. Network Sci.

[17] Onaga T, Gleeson JP, Masuda N (2017). Concurrency-induced transitions in epidemic dynamics on temporal networks. Phys Rev Lett.

[18] Salathé M, Kazandjieva M, Lee JW, Levis P, Feldman MW, Jones JH (2010). A high-resolution human contact network for infectious disease transmission. Proc Natl Acad Sci.

[19] Gleich DF (2015). PageRank beyond the web. SIAM Rev.

[20] Easley D, Kleinberg J (2010). Networks, crowds, and markets: Reasoning about a highly connected world.

[21] Yang Y, Hao F, Park D, Peng S, Lee H, Mao M (2021). Modelling prevention and control strategies for COVID-19 propagation with patient contact networks. IEEE Access.

[22] Materassi D, Salapaka MV. Identification of network components in presence of unobserved nodes. 2015 54th IEEE Conference on Decision and Control (CDC). 2015;1563–1568. 10.1109/cdc.2015.7402433.

[23] Faust K (2010). A puzzle concerning triads in social networks: Graph constraints and the triad census. Soc Networks.

[24] Pinter-Wollman N, Hobson EA, Smith JE, Edelman AJ, Shizuka D, de Silva S, Waters JS, Prager SD, Sasaki T, Wittemyer G (2014). The dynamics of animal social networks: analytical, conceptual, and theoretical advances. Behav Ecol.

[25] Memic H, Husagic-Selman A, Hadziabdic K, Triadic patterns of friendships in YouTube groups. 2011 IEEE 12th International Symposium on Computational Intelligence and Informatics (CINTI), vol. 2011. IEEE. p. 501–6. https://ieeexplore.ieee.org/document/6108558

[26] Boccaletti S, Ivanchenko M, Latora V, Pluchino A, Rapisarda A (2007). Detecting complex network modularity by dynamical clustering. Phys Rev E.

[27] Que X, Checconi F, Petrini F, Gunnels JA (2015). Scalable community detection with the louvain algorithm.

[28] Barabási A, Albert R (1999). Emergence of scaling in random networks. Science.

[29] Lau MS, Dalziel BD, Funk S, McClelland A, Tiffany A, Riley S (2017). Spatial and temporal dynamics of superspreading events in the 2014–2015 West Africa Ebola epidemic. Proc Natl Acad Sci.

[30] Hu Y, Guo J, Li G, Lu X, Li X, Zhang Y, Cong L, Kang Y, Jia X, Shi X, Xie G, Zhang L (2021). Role of efficient testing and contact tracing in mitigating the COVID-19 pandemic: a network modelling study. BMJ Open.

